# Modeling of α/β for late rectal toxicity from a randomized phase II study: conventional versus hypofractionated scheme for localized prostate cancer

**DOI:** 10.1186/1756-9966-28-117

**Published:** 2009-08-19

**Authors:** Simona Marzi, Biancamaria Saracino, Maria G Petrongari, Stefano Arcangeli, Sara Gomellini, Giorgio Arcangeli, Marcello Benassi, Valeria Landoni

**Affiliations:** 1 Laboratorio di Fisica Medica e Sistemi Esperti, Istituto Regina Elena, Rome, Italy; 2 S.C. Radioterapia, Istituto Regina Elena, Rome, Italy

## Abstract

**Background:**

Recently, the use of hypo-fractionated treatment schemes for the prostate cancer has been encouraged due to the fact that α/β ratio for prostate cancer should be low. However a major concern on the use of hypofractionation is the late rectal toxicity, it is important to be able to predict the risk of toxicity for alternative treatment schemes, with the best accuracy. The main purpose of this study is to evaluate the response of rectum wall to changes in fractionation and to quantify the α/β ratio for late rectal toxicity

**Methods:**

162 patients with localized prostate cancer, treated with conformal radiotherapy, were enrolled in a phase II randomized trial. The patients were randomly assigned to 80 Gy in 40 fractions over 8 weeks (arm A) or 62 Gy in 20 fractions over 5 weeks (arm B). The median follow-up was 30 months. The late rectal toxicity was evaluated using the Radiation Therapy Oncology Group (RTOG) scale. It was assumed ≥ Grade 2 (G2) toxicity incidence as primary end point. Fit of toxicity incidence by the Lyman-Burman-Kutcher (*LKB*) model was performed.

**Results:**

The crude incidence of late rectal toxicity ≥ G2 was 14% and 12% for the standard arm and the hypofractionated arm, respectively. The crude incidence of late rectal toxicity ≥ G2 was 14.0% and 12.3% for the arm A and B, respectively. For the arm A, volumes receiving ≥ 50 Gy (V50) and 70 Gy (V70) were 38.3 ± 7.5% and 23.4 ± 5.5%; for arm B, V38 and V54 were 40.9 ± 6.8% and 24.5 ± 4.4%. An α/β ratio for late rectal toxicity very close to 3 Gy was found.

**Conclusion:**

The ≥ G2 late toxicities in both arms were comparable, indicating the feasibility of hypofractionated regimes in prostate cancer. An α/β ratio for late rectal toxicity very close to 3 Gy was found.

## Background

During the last years a wide consensus has been growing on the fact that α/β ratio for prostate cancer should be low [1-6], encouraging the use of hypo-fractionated treatment schemes. This would result in an increased therapeutic ratio besides a well known series of practical advantages, like diminishing the number of accesses to department, shorter treatment time and abatement of waiting lists. Due to the fact that a major concern on the use of hypofractionation is the late rectal toxicity, the necessity to predict the risk of toxicity for alternative treatment schemes is becoming insistent. Leborgne [7], in a study conducted on patients treated with brachytherapy for cancer of the cervix, evaluated an α/β ratio for rectal late complications not significantly different from 3 Gy. In a more recent publication, Brenner [8] underlined the importance of investigating the sensitivity of late rectal damage to changes in fractionation and encouraged the use of new data from hypofractionated schemes. His analysis resulted in an α/β ratio estimate of 5.4 Gy, suggesting a correlation with early-responding damage.

Since 2003, a phase II randomized trial started at our institute, to compare a conventional versus a hypofractionated treatment scheme for localized prostate cancer. It was assumed an α/β ratio for prostate of 1.5 Gy. The primary objective of the trial were acute and late toxicity, and survival and local control with controlled PSA (Prostate Specific Antigen). In this work, dose-volume data of rectal wall from patients treated exclusively at our institution were fitted to the Normal Tissue Complication Probability (NTCP) model proposed by Lyman-Kutcher-Burman [9-11]. The effect of dose fractionation was included in the model to quantify the α/β ratio for late rectal toxicity.

## Methods

### Patient population

From March 2003 to June 2008, 162 patients with carcinoma of the prostate were randomised for the present study. Assuming that an incidence of ≥ Grade 2 (G2) toxicity in less than 55% of patients is acceptable, the sample size was calculated for a power of 80% and a level of significance of 5%. A total of 114 patients, having a follow-up longer than 6 months, were included in the present analysis: 57 patients in each arm. All patients enrolled in this trial were younger than 85 with high risk prostatic carcinoma, that is at least two of the following risk factors present: T2c-T4, PSA > 10 ng/ml, Gleason score 7-10. Other eligibility criteria were no nodes involvement present at Computer Tomography (CT) or Magnetic Resonance imaging, no other previous radiotherapy (RT) or prostatectomy, no other malignant disease except for Basal cell carcinoma (BCC) or other tumors in the past five years, informed consent.

Patients received hormonal treatment (HT), in addition to RT, two months before; Casodex (non-steroidal anti-androgen) was administered for 270 days, Zoladex (analogous Goserelin) was started 7 days after the start of Casodex and was administered at the 7^th^, 97^th ^and 187^th ^day.

The clinical and pathological features of the two groups of patients are reported in Table 1. The baseline recorded characteristics were age, initial PSA values (≤ 10, between 11 and 20 and > 20 ng/mL), stage (<T2c vs. ≥ T2c), and Gleason score (≤ 6 vs. > 6). The differences between groups were tested using chi-square.

**Table 1:** Clinical and pathological features of the two patients populations

Characteristics	Arm A	Arm B	p value
Age			0,922
< 70	8	7	
71-75	23	22	
> 75	26	28	
Stage			1,000
<T2c	27	26	
≥ T2c	30	31	
Gleason Score			0,392
≤ 6	9	5	
> 6	48	52	
initial PSA			0,400
≤ 10	18	14	
11-20	20	17	
> 20	19	26	

### Contouring, planning and treatment

The clinical target volume (CTV) was the prostatic gland and the seminal vescicles; the planning target volume (PTV) was obtained by expanding CTV with a margin of 1 cm in each direction, and of 0.6 cm posteriorly. Rectum was manually contoured from the distal ischiatic branch to the sigmoid flexure as a hollow organ, i.e. rectal wall. In addition bladder wall and femoral heads were contoured.

Dose calculations were performed using the treatment planning system Eclipse (Release 6.5, Varian Associates, Palo Alto, CA), to deliver the prescribed dose to the International Commission on Radiation Units and Measurements (ICRU) reference point [12], with a minimum dose of 95% and a maximum dose of 107% to the PTV.

Dose-volume constraints on rectal wall were: no more than 30% of rectal wall receiving more than 70 Gy (V_70_) and no more than 50% of rectal wall receiving more than 50 Gy (V_50_) for the conventional arm; no more than 30% of rectal wall receiving more than 54 Gy (V_54_) and no more than 50% of rectal wall receiving more than 38 Gy (V_38_) for the hypo-fractionated arm. Dose-volume constraints on bladder wall were: V_70 _less than 50% for the conventional arm and V_54 _less than 50% for the hypo-fractionated arm. Maximum dose on femoral head was, whenever achievable, less than 55 Gy and 42 Gy for arm A and arm B, respectively. Safer dose volume constraints in the hypofractionation arm were intentionally chosen; that is as if the equivalence was calculated with an α/β value lower than 3 Gy.

Treatment plans were designed with a 3DCRT (three dimensional conformal radiation therapy) six field technique, with gantry angles: 45°, 90°, 135°, 225°, 270°, 315°. The two posterior-oblique fields had 45° wedges and all fields were conformed with a multileaf collimator (MLC). Treatments were delivered with 15 MV photon beam generated by a Clinac 2100 CD Varian accelerator, equipped with Millennium MLC (120 leaves).

### Toxicity evaluation

Rectal toxicity was assessed using the Radiation Therapy Oncology Group (RTOG) scale [13], every six months for the first three years after the end of treatment and afterwards every year. The incidence of ≥ G2 late rectal toxicity as a function of time (months from the end of treatment) was evaluated by Kaplan-Meier curves using MedCalc software (Version 8.1.0.0, Mariakerke, Belgium). The log rank test was performed to establish if any statistically significant difference exists between the two arms.

### Radiobiologic calculations

Cumulative dose-volume histograms (DVHs) have been first evaluated for the two arms, independently. Then, to compare the two different treatment schemes, DVHs for both arms have been corrected converting the physical dose in the i-th volume fraction to the biologically equivalent total dose normalized to the standard fraction of 2 Gy (NTD_2_), as described in appendix 1 (A.5).

The Lyman-Burman-Kutcher (*LKB*) model was used to predict the NTCP for late rectal toxicity. The ≥ G2 late rectal toxicity was assumed as primary end point in the NTCP calculations. The original model parameters are n, m and TD_50 _and they determine the volume dependence of NTCP, the slope of NTCP vs. dose and the tolerance dose to the whole organ leading to a 50% complication probability, respectively (appendix 1). The α/β parameter was then introduced in the model by the NTD_2 _to take into account for altered fractionaction schemes, as illustrated also by other authors [14,15].

At first, the values n = 0.12, m = 0.15 estimated by Burman *et al*. [10] and the value TD_50 _= 80 Gy evaluated by Emami *et al*. [16] were involved in the calculation of the NTCP distributions for conventional and hypofractionated arms.

To minimize the deviation between the clinical and the predicted complication incidences, the best parameters estimation of the model was performed by the maximum likelihood method [17]. For binomially distributed data such as the NTCP data, the log-likelihood for the entire data set is given by:



where N is the total number of patients, *R*_*i *_is equal to 1 for patients who did experience ≥ G2 late rectal toxicity or 0 for patients who did not.

The optimization of all the four model parameters was initially run but, because of the large resulting 95% confidence intervals (CI) due to the limited number of patients experiencing ≥ G2 late toxicity, the results were not reported. Consequently, it was decided to reduce the number of degrees of freedom by keeping fix the n and m parameters at the original values proposed by Burman *et al*. [10]. This choice was motivated by the fact that these values, even if obtained assuming as end point severe proctitis, necrosis, stenosis or fistula, resulted hardly different from those reported in more recent studies of late rectal toxicity modeling [18,19], in which similar end points to that considered in the present work were assumed. Moreover, this choice is in accordance with our belief that rectal bleeding is most strongly influenced by high dose levels (low n value) [20].

The 95% CI of the estimated TD_50 _and α/β parameters were established by the profile likelihood method as described by other authors [21]. All the calculations were performed by using the Matlab code (Release 6.5, The Mathworks Inc., Natick, Massachusetts).

## Results

### DVH analysis

**Figure 1:**
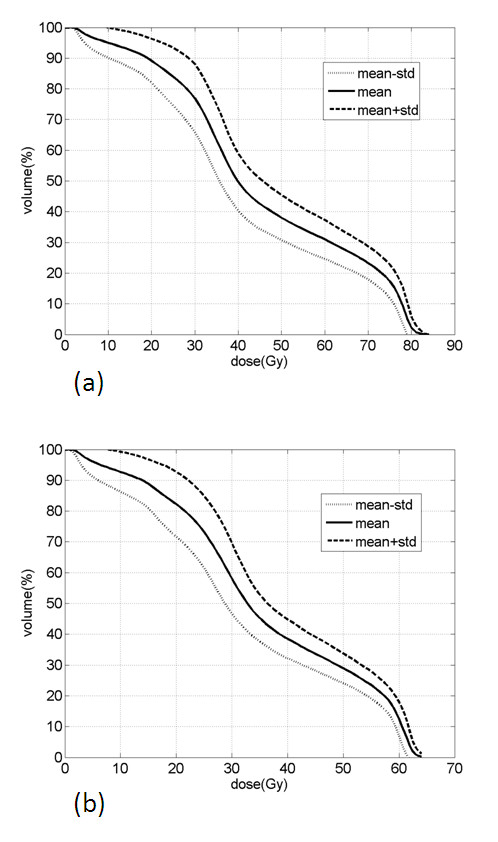
**(a) The average with its standard deviation of the distribution of the cumulative rectal wall DVHs for the conventional arm**. (b) The average with its standard deviation of the distribution of the cumulative rectal wall DVHs for the hypofractionated arm.

Differential and cumulative dose-volume histograms of each patient were collected. For both arms dose-volume constraints were well satisfied: for arm A, V_50 _and V_70 _resulted 38.3 ± 7.5% and 23.4 ± 5.5%, respectively; for arm B, V_38 _and V_54 _resulted 40.9 ± 6.8%. and 24.5 ± 4.4%, respectively (Fig. 1). From the small standard deviation of V_50_/V_70 _and V_38_/V_54_, it can be inferred that all patients were almost equally treated among each arm with respect to the dose distribution of the rectal wall.

**Figure 2:**
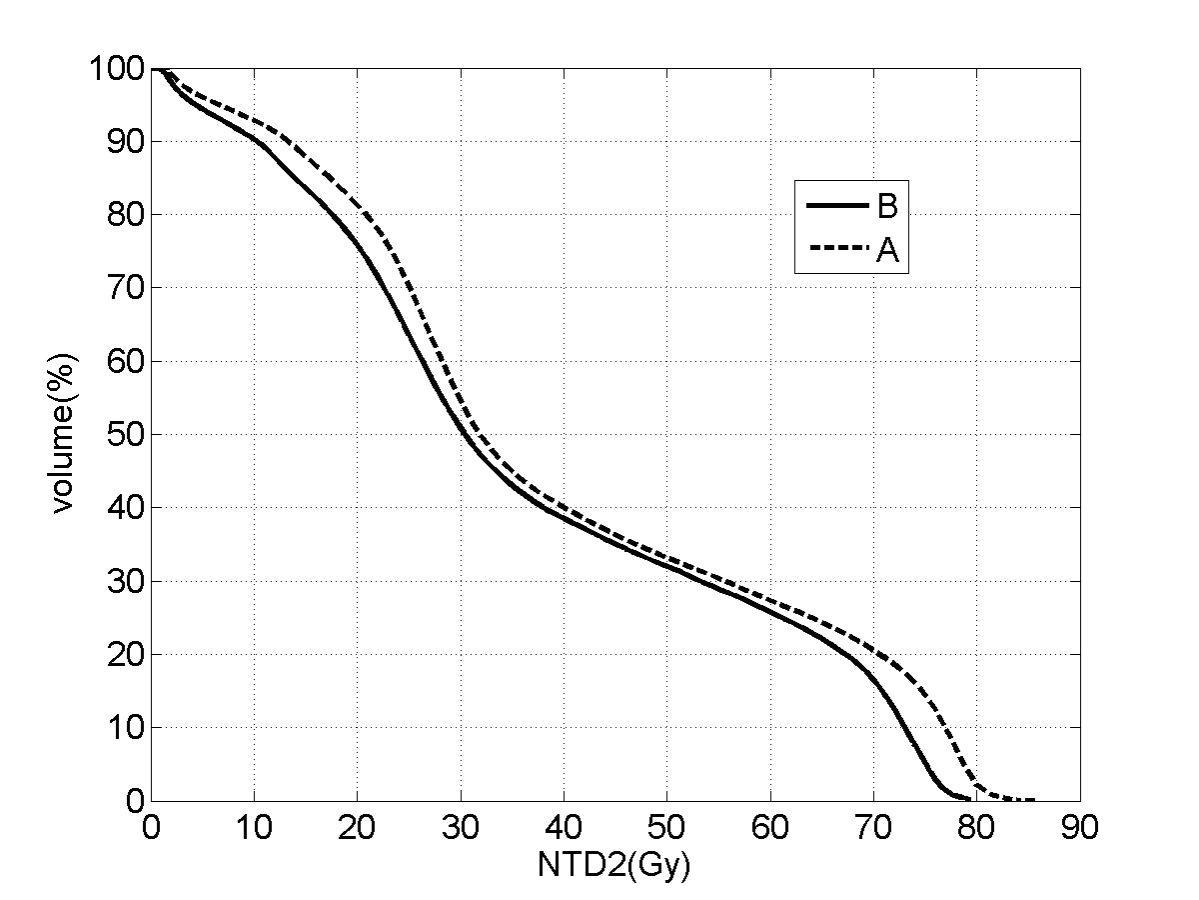
**The averages of the distributions of the normalized cumulative rectal wall dose-volume-histograms for arm A (dashed line) and for arm B (solid line)**. NTD_2 _on the X-axis indicates the biologically equivalent total dose normalized to the standard fraction of 2 Gy, supposing an α/β ratio of 3 Gy.

To compare the two different treatment schemes, DVHs for the two arms have been both normalized, converting the physical dose in each volume fraction to the NTD_2 _(A.5) supposing an α/β ratio of 3 Gy. The plot in Fig. 2 shows together the corrected DVHs for the two arms: the two curves are very close to each other, suggesting the equivalence of the conventional and the hypofractionated schemes in terms of the expected ≥ G2 late rectal toxicity.

### Incidence of late toxicity

**Figure 3:**
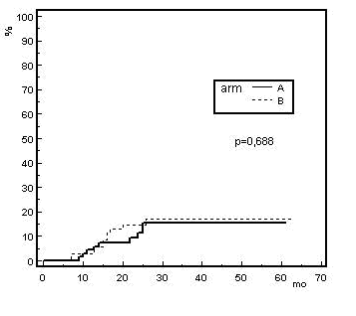
**Actuarial incidence of ≥ Grade 2 late rectal toxicity versus months after radiotherapy (mo.), for arm A and B**.

The crude incidence ≥ G2 late rectal toxicity was 14.0% (8 patients) and 12.3% (7 patients) for the conventional and the hypo-fractionated arm respectively, after a median follow up of 30 months for both arms (range: 6-61 months for arm A, 6-63 months for arm B). In arm A, three patients experienced G3 toxicity and no patient developed G4; while in arm B no patients had late toxicity higher than G2. The actuarial ≥ G2 late toxicity at 30 months were 13.0% and 13.5% for arm A and B, respectively, as illustrated by the Kaplan-Meier curves in Fig. 3. No significant difference exists between the curves (p-value = 0.688 by the log rank test).

### NTCP modeling: optimization of TD_50 _and α/β

NTCP distributions were calculated for both arms to estimate the probability of ≥ G2 late rectal toxicity, using the values n = 0.12, m = 0.15, TD_50 _= 80 Gy and α/β = 3 Gy. An average probability of 9.6% ± 3.3% and 5.6% ± 1.8% were obtained for the conventional and the hypo-fractionated arm, respectively.

**Figure 4:**
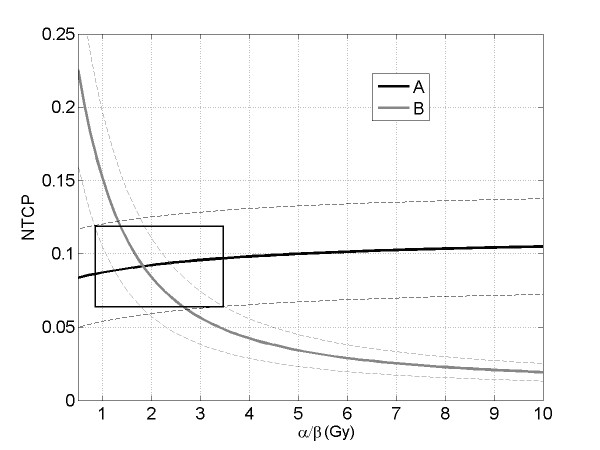
**Plot of the average Normal Tissue Complication Probability (NTCP) with its standard deviation (dashed lines) versus the α/β parameter, for the arm A (black line) and B (gray line)**. The other parameters were n = 0.12, m = 0.15 and TD_50 _= 80 Gy. The width of the box indicates the range of probable α/β values.

These NTCP calculations did not result in good agreement with the clinical outcome for both arms, indicating the necessity to optimize the model parameters. Before the modeling, a plot of NTCP with its standard deviation versus α/β was generated for the arms A and B to better evaluate the influence of α/β on the toxicity prediction (Fig. 4). The plotted NTCP values were obtained by averaging on the entire patients population of arm A and B, separately, the NTCP data calculated varying α/β between 0.5 and 10 Gy, at 0.1 Gy intervals. The other three parameters were kept fix (n = 0.12, m = 0.15, TD_50 _= 80 Gy).

As expected, it resulted that higher values of α/β lead to an increase of NTCP in arm A, because the effect of fractionation (or the dose per fraction) weights less that the effect of the total dose. For the same reason, the NTCP in arm B rapidly decreases at increasing values of α/β, because the total dose of the hypofractionated arm (62 Gy) is expected to induce a significantly lower complication than the total dose of the conventional arm (80 Gy). Due to the comparable toxicities reported among the two arms, it is meaningful to observe the plots in the region where the two NTCP curves overlap. Also taking into account the NTCP standard deviations, the plots suggest approximately an α/β value between 1 and 3.5 Gy (given by the width of the box), with a most probable value close to 2 Gy (where the average NTCP values are coincident).

Together with α/β, the parameter TD_50 _was also optimized because, as previously observed, the complication incidence predicted by the model using TD_50 _= 80 Gy was lower than the clinical outcome for both arms (9.6% and 5.6% against 13.0% and 13.5%, for arm A and B respectively). The m and n parameters were kept fix during the modeling, choosing the values: n = 0.12 and m = 0.15 (10), as mentioned in the Methods and materials.

The value of TD_50 _was decreased by the fitting process, resulting equal to 76.0 Gy [95% CI: 72.2-80.5 Gy]. The best estimate for α/β was instead 2.3 Gy [95% CI: 1.1-5.6 Gy]. To evaluate the goodness of fit, the observed and expected numbers of complications (or events) were compared for six NTCP groups (Table 2).

**Table 2:** Observed and expected numbers of complications in six NTCP groups

NTCP range	No. of patients	Observed Complications	Expected Complications
0.05-0.075	11	2	1
0.075-0.10	19	3	2
0.10-0.125	18	3	2
0.125-0.15	25	2	4
0.15-0.175	27	4	4
0.175-0.25	15	1	3

The chi-squared value, obtained as the sum of the squares of differences between the observed and expected numbers of complications divided by the expected numbers of complications, resulted 4.3. Being the chi-square value 11.07 for 5 degrees of freedom and a 5% significance level, it cannot be rejected the hypothesis that the fit is acceptable. NTCP values have been recalculated for the two arms with the optimized parameters; the values of clinical incidence fall now inside the confidence intervals of NTCP, as shown in Table 3.

**Table 3:** Clinical incidence of ≥ G2 late toxicity and NTCP calculations

	A	B
Clinical incidence	14.0%	12.3%
NTCP (prior to optimization) TD_50 _= 80 Gy, α/β = 3 Gy	10 ± 3%	6 ± 2%
NTCP (after optimization) TD_50 _= 76 Gy, α/β = 2.3 Gy	15 ± 5%	12 ± 4%

## Discussion

In this work, a modeling of late rectal toxicity in patients with localized prostate cancer was performed. The patients were randomly assigned to receive 80 Gy in 40 fractions over 8 weeks (arm A) or 62 Gy in 20 fractions over 5 weeks to the prostate (arm B). The comparison between the conventional and the hypofractionated arms allowed to evaluate the response of rectal toxicity to changes in fractionation.

The crude rate of ≥ G2 late rectal toxicity were 14.0% and 12.3% for arm A and B respectively, thus very close to the actuarial values at 30 months (Fig. 3), indicating that this time can be considered adequate to report the late rectal toxicity, as documented also by other studies [18,22,23]. The comparable toxicity rates observed in the two arms suggest that the hypofractionated regimes in prostate cancer are feasible, as previously reported in other studies [24-29], though using different fractionation schemes and end point definitions. Lukka *et al*. [24] compared two fractionation schemes for patients with localized prostate cancer, in a randomized trial designed to give 66 in 33 fractions or 52.5 Gy in 20 fractions to the prostate. The authors reported similar ≥ G3 late rectal toxicity incidence in both arms (1.3%), with a long median follow-up time of 5.7 years. Livsey *et al*. [26] also analyzed bowel toxicity in hypofractionated regime, giving to the prostate 50 Gy in 16 fractions. The reported ≥ G2 bowel toxicity was lower (5%), presumably due to the consistently lower total dose.

Among all studies, the present work is best comparable to the study of Faria *et al*. [29], who analyzed late rectal toxicity in prostate cancer patients receiving 66 Gy in 22 fractions. They reported a crude incidence of ≥ G2 late rectal toxicity of 18%, with a median follow-up time of 30 months. The deviation from our rate of toxicity probably arise from the different total dose (66 against 62 Gy). Assuming to prescribe to our patients of arm B 66 Gy in 22 fractions to the PTV, with the same relative dose distribution to the rectal wall, the average NTCP would result 17.5 ± 4.8% with our best-fit parameters. This calculation is in good agreement with the crude toxicity of 18% of the study of Faria *et al*.[29].

The present work was undertaken with the main purpose of quantifying the α/β ratio for ≥ G2 late rectal damage, that still represents the dose limiting end point in prostate radiotherapy. The rectum has been defined as rectal wall, instead of the total rectal volume including filling, allowing to improve the fit accuracy as suggested by others [21]. It was found that the best estimation for TD_50 _is 76.0 Gy [72.2-80.5 Gy], a value slightly lower than the value of 80 Gy of Emami *et al*. [16] and also in agreement with a more recent estimate proposed by Peeters *et al*. [19], who found TD_50 _= 81 Gy (68% CI = 75-90 Gy) for the same end point and a minimum follow-up time of 3 years.

The estimated α/β = 2.3 Gy [95% CI: 1.1-5.6 Gy] is consistent with the interval of α/β values suggested by the plot of NTCP versus the α/β ratio illustrated in Fig. 4 and is also consistent with the initial supposed value of 3 Gy. In fact, assuming α/β = 3 Gy it was shown the equivalence of the normalized cumulative rectal wall DVHs of the two arms (Fig. 2), that suggested comparable expected toxicities as then confirmed by our outcome data.

A value of α/β close to 3 Gy is also in accordance with the conclusions of a study of Leborgne *et al*. [7], who performed calculations of Biologically Effective Doses (BEDs) in medium dose rate brachytherapy of cervix cancer. The authors stated that assuming α/β equal to 3 Gy for rectal late responding tissues seems to be a provisional value that may be of use in comparing the expected effects of new schedules. This estimate is indeed more distant from that one given by Brenner [8] (5.4 ± 1.5 Gy), who made a fit of late rectal toxicity data coming from four different institutions, with doses per fraction between 1.8 and 3 Gy. This value, between typical α/β values for early and late-responding tissues, would suggest that the late rectal damage could be correlated with the very acute one, in accordance with conclusions of other studies [30-32]. The discrepancy between these α/β estimates might be due to differences in the underlying data. However, as documented by the literature [33] it is a matter of debate whether there is a real causative relationship between acute and late rectal reactions and the question is still open.

In the present analysis, it was decided not to take into account the effect of rectal motion. In fact, a previous study of our group [34] was conducted on patients treated for prostate cancer with IMRT. The average NTCP values showed a small variation during the radiation treatment, if compared to those obtained from the original plan optimized on the pre-treatment CT: 7.2% ± 2.9% versus 6.7% ± 2.1%, respectively. Moreover, it is reasonable to assume that in 3DCRT these variations might be even smaller than in IMRT, due to the less steep dose gradients across the rectum.

## Conclusion

In this work, a modeling of late rectal toxicity in patients with localized prostate cancer, from a randomized phase II study, was performed. The comparison between the conventional and the hypofractionated arm allowed to evaluate the response of rectal toxicity to changes in fractionation. The similar rate of late toxicity in the two arms seems to indicate the feasibility of hypofractionated regimes in prostate cancer. Our study led to an estimation of α/β ratio value for late rectal toxicity very close to 3 Gy; however further prospective studies need to be performed to definitely establish the value of the α/β ratio in a larger cohort of patients enhancing the accuracy of the radiobiological modeling.

## Competing interests

The authors declare that they have no competing interests.

## Authors' contributions

SM, GA, MB and VL conceived of the study and partecipated in its design and coordination. BS, MGP, SG and SA contributed with the enrollement of patients, were responsible of the radiotherapy treatments and collected the patient's clinical data. SM and VL performed the radiobiological modelling and the statistical analyses, and wrote the manuscript. All authors read and approved the final draft.

## Appendix 1

For the *LKB *model [9,10], assuming a uniform irradiation of a fraction *v *of the organ at dose *D*, NTCP can be calculated by

(A.1)

where *t *is defined as

(A.2)

and

(A.3)

As known, the parameters n, m and TD50(1) determine the volume dependence of NTCP, the slope of NTCP vs. dose and the tolerance dose to the whole organ leading to a 50% complication probability, respectively.

The effective volume method [11] was chosen as histogram reduction scheme for non uniform organ irradiations:

(A.4)

where *D*_*i *_is the dose delivered to the volume fraction *v*_*i *_and *N *is the number of points of the differential DVH. By (A.4), an inhomogeneous dose distribution is converted to an equivalent uniform irradiation of a fraction *v*_*eff *_of the organ at the maximum dose *D*_*max*_. Before applying the above equations, a correction is performed to *D*_*i*_, to take into account the fractionation inside each volume fraction *v*_*i*_. In this way, the physical dose *D *in each volume fraction *v *is converted to the biologically equivalent total dose normalized to the standard fraction of 2 Gy (NTD_2_).

(A.5)

where the parameters α and β are the coefficients of the linear and quadratic dose contributions to damage in the linear-quadratic model of the cell survival curve and *n*_*fr *_is the number of fractions.
